# Effects of Forage Source and Method of Offering on Growth Performance, Starter Feed Intake, Rumen Fermentation, and Selected Blood Parameters in Preweaned Holstein Calves

**DOI:** 10.3390/ani16101462

**Published:** 2026-05-10

**Authors:** Hande Cansu Başaran, İbrahim İsmet Türkmen

**Affiliations:** Department of Animal Nutrition and Nutritional Diseases, Faculty of Veterinary Medicine, Bursa Uludag University, Görükle Campus, 16059 Nilüfer, Bursa, Türkiye

**Keywords:** Holstein calf, preweaning period, forage source, alfalfa hay, straw, starter feed, rumen fermentation, growth performance, fecal score, beta-hydroxybutyrate

## Abstract

This study evaluated the effects of forage source and method of offering on growth performance, starter feed intake, fecal score, rumen fermentation, and selected blood parameters in preweaned Holstein calves. Forty calves were assigned to four treatments (*n* = 10/group): straw offered separately from starter feed (A1), starter feed containing 7% straw (A2), alfalfa hay offered separately from starter feed (A3), or starter feed containing 7% chopped alfalfa hay (A4). Calves were monitored from birth to 60 days of age. On day 60, body weight was higher in A3 than in A1 (73.37 ± 7.87 vs. 65.82 ± 6.41 kg; *p* = 0.030). Average daily gain was higher in A3 than in A1 during days 15–30, higher in A3 and A4 than in A1 during days 30–45, and higher in A2 and A3 than in A1 during days 45–60 (*p* < 0.05). Starter feed intake differed during weeks 5–7, with higher values mainly observed in the straw-fed groups (*p* < 0.001). Fecal score was higher in A4 than in A2 in week 2 and higher in A4 than in A1 during weeks 3–6 (*p* < 0.05). Rumen pH on day 60 was higher in A4 than in A1 and A3 (*p* = 0.003), whereas volatile fatty acid concentrations did not differ among groups. Serum glucose on day 60 was higher in A3 than in A1 (*p* = 0.002), and total protein was lower in A1 than in A2, A3, and A4 on day 60 (*p* < 0.001). These findings suggest that forage source and method of offering may produce different biological responses in preweaned calves.

## Simple Summary

The preweaning period is a critical stage for calf growth, digestive development, and adaptation to solid feed. In this study, straw or alfalfa hay was offered to Holstein calves either separately from starter feed or mixed with starter feed. Offering alfalfa hay separately from starter feed resulted in a more favorable final body weight than offering straw separately. In contrast, mixing chopped alfalfa hay with starter feed increased rumen pH, suggesting a possible improvement in rumen buffering conditions. Starter feed intake and growth performance did not always change in the same direction, indicating that forage quality and method of offering should be considered together. These findings suggest that alfalfa hay may be more advantageous than straw during the preweaning period, but the preferred method of offering may depend on whether the main goal is to support growth performance or rumen buffering.

## 1. Introduction

The preweaning period is a critical stage in calf development, during which calves transition from liquid feeding to solid feed, rumen development accelerates, and metabolic adaptation is established [1,2]. Nutritional management during this period may affect not only body weight gain, but also starter feed intake, rumen fermentation, selected blood parameters, and digestive health [1,3]. Feeding strategies applied early in life may influence not only short-term growth performance, but also later productive potential and physiological responses [4,5].

Early forage provision may play a role in adaptation to solid feed by affecting rumination behavior and feed consumption patterns [6]. The structural and functional development of the rumen accelerates with the onset of solid feed intake. Increased solid feed consumption promotes the production of volatile fatty acids, particularly butyrate and propionate, which support ruminal epithelial development and functional capacity. Volatile fatty acids produced in the rumen are important for the development of ruminal papillae and the enhancement of absorptive capacity [2,3]. Starter feed intake is considered one of the main determinants of rumen development [7,8]. During this period, not only the amount of starter feed intake, but also the presence or absence of forage in the diet, the forage source, and the method by which forage is offered may influence rumen conditions and calf performance [3,9]. Changes in the fiber level and physical characteristics of starter diets may also exert different effects on growth performance, rumen fermentation, and selected developmental parameters [10,11].

Forage feeding has been reported to influence chewing and rumination behavior, salivary secretion, rumen pH, and the digestive process in calves [3,12]. A simple “forage” versus “no forage” approach may therefore be insufficient, and the nutrient composition, physical form, and method of offering forage, either separately or together with starter feed, should also be considered [9,13]. Omidi-Mirzaei et al. [13] reported that forage source and particle size affected growth performance, rumen fermentation, and behavior. Engelking et al. [9] also showed that offering forage separately from starter feed or as a mixture could alter feed intake and growth rate. Straw and alfalfa hay were selected because they represent forage sources with contrasting nutrient composition and physical characteristics. In the present study, alfalfa hay had higher crude protein content and lower fiber concentration than straw, whereas straw had a higher structural fiber content and lower nutritive value. These differences were expected to influence not only nutrient supply, but also feed intake behavior and rumen conditions. The method of offering was also expected to be important. A separate offering may allow calves to regulate forage intake according to individual preference, whereas mixing chopped forage with starter feed may promote more simultaneous consumption of structural fiber and concentrate. This could affect chewing activity, salivary buffering, and rumen pH.

Reported effects of forage provision have not always been consistent. Omidi-Mirzaei et al. [13] found that calves receiving alfalfa hay had greater starter intake and higher weaning body weight during the preweaning period. Antúnez-Tort et al. [14], who compared barley straw and alfalfa hay, found no difference in total dry matter intake before weaning. These findings suggest that the effects of forage source may vary according to the method of application, the physical characteristics of the forage, and the timing of forage provision. In different studies, the effects of forage supplementation on starter intake and growth performance have been reported as positive, neutral, or limited, depending on factors such as the physical form of starter feed, forage type, and calf age [15,16].

Forage provision has also been associated with changes in rumen pH. Kim et al. [3] reported higher mean and maximum rumen pH values in calves receiving forage-containing diets, whereas Bennett et al. [12] found that lower forage levels reduced rumen pH. Changes in rumen pH do not always correspond to similar changes in volatile fatty acid profiles [12,13]. Alterations in rumen conditions do not always directly align with fermentation end products and performance traits, which reflects the multifactorial nature of these processes [7].

Several studies have examined forage source, particle size, or method of offering in preweaned calves. However, the biological responses to forage provision may differ depending on whether the forage is evaluated as a source effect, an offering-method effect, or a combined feeding strategy. The present study was designed to compare straw and alfalfa hay offered either separately from starter feed or mixed with starter feed under the same experimental conditions. The aim was to evaluate not only growth performance and starter feed intake, but also fecal score, rumen pH, volatile fatty acids, and selected blood parameters. Rather than claiming absolute novelty, the contribution of this study is the integrated comparison of forage source and offering method and the interpretation of their distinct responses in growth performance and rumen conditions.

## 2. Materials and Methods

### 2.1. Animals, Experimental Site, and Experimental Design

This study was conducted at the Ömer Matlı Livestock Application and Research Center located in Karacabey, Bursa, Türkiye. A total of 40 Holstein calves were used. Calves were assigned to the treatment groups sequentially according to birth order as they entered the study, while maintaining a sex-balanced distribution among groups. Each group included 5 male and 5 female calves to minimize potential sex-related imbalance among treatments. Birth weight was recorded but was not used as a blocking factor during allocation. Calves were housed in individual outdoor hutches throughout the experimental period. The animals were assigned to four treatment groups, with 10 calves in each group. In group A1, starter feed and straw were offered separately, with straw provided ad libitum. In group A2, straw was mixed into the starter feed at 7%. In group A3, starter feed and alfalfa hay were offered separately, with alfalfa hay provided ad libitum. In group A4, chopped alfalfa hay was mixed into the starter feed at 7%. The individual calf was considered the experimental unit. The study protocol was approved by the Local Ethics Committee for Animal Experiments of Bursa Uludag University (UÜHADYEK; decision no. 2022-06/02; 19 April 2022).

### 2.2. Feeding and Management

The calves were fed colostrum during the first three days of life. Colostrum feeding was initiated within 1–2 h after birth, and the amount offered was planned to be approximately 10% of body weight. A second colostrum meal was provided within the first 12 h, and total colostrum intake during the first 24 h was approximately 6 L. From day 3 onward, the calves received the solid feed treatments assigned to their groups and had free access to drinking water. In the mixed-forage treatments, straw and alfalfa hay were chopped before incorporation into the starter feed. The 7% inclusion level used in the mixed-forage treatments was selected to provide a moderate amount of structural fiber while avoiding excessive dilution of the nutrient density of the starter feed. The same inclusion level was used for straw and chopped alfalfa hay to allow for a comparison of forage source under a controlled mixed-offering condition. The chopped straw used in group A2 and the chopped alfalfa hay used in group A4 were prepared to an approximate nominal chop length of 1–2 cm. Geometric mean particle size was not determined using a standardized particle-size method, such as dry sieving or the Penn State Particle Separator; therefore, particle size is reported as an approximate physical description of the forages used in the experiment. All calves were fed a milk replacer (Kalvolac Robust Calf). The milk replacer was prepared according to the manufacturer’s recommendations and offered at a total of 5 L/day, consisting of 2 L in the morning, 2 L in the evening, and 1 L at night. All calves received milk replacer throughout the 60-day experimental period and were not weaned during the trial. Day 60 was considered the final day of data collection. After completion of the experimental period, calves underwent a gradual 5-day weaning program as part of routine farm management. During the first 2 days of this post-experimental weaning program, milk replacer was offered twice daily, whereas during the final 3 days, it was offered once daily. Calves were fully weaned at the end of this 5-day program. Therefore, weaning was not included as an experimental factor, and all measurements reported in this study were obtained before weaning. The nutrient composition of the milk replacer, starter feed, and forages used in the experiment is presented in Table 1 and Table 2.

### 2.3. Growth Performance, Starter Feed Intake, and Fecal Score

Body weight was measured on days 0, 15, 30, 45, and 60. Weekly starter feed intake was recorded, and average daily gain values were calculated for the periods between weighings. Individual forage intake was not recorded in calves receiving straw or alfalfa hay separately. Although calves were housed in individual outdoor hutches, environmental factors such as wind, rain, wetting, and feed spillage made reliable measurement of forage refusals difficult. Therefore, only starter feed intake was used for intake-related analyses, and total dry matter intake could not be calculated for the separately offered forage groups. Starter feed intake was calculated as the difference between the amount of feed offered and the amount remaining at the next measurement. Fecal scores were evaluated weekly from birth to day 60 by the same researcher using visual inspection. The scoring system developed by Pennsylvania State University, ranging from 1 to 5, was used [17]. In the present study, the scores were defined as follows: 1 = firm/normal feces, 2 = soft feces with a distinct form, 3 = soft feces with no clear form, 4 = watery feces, and 5 = watery feces containing blood and/or mucus.

### 2.4. Blood Sampling and Analyses

Blood samples were collected twice from each calf, at 24–36 h after birth and on day 60, approximately 3–4 h after feeding, from the jugular vein using a vacuum blood collection system (BD Vacutainer, Becton Dickinson, Franklin Lakes, NJ, USA). For each calf, blood was collected into two 10 mL serum tubes. The samples were centrifuged at 10,000 rpm for 3–4 min to separate the serum. The serum was then transferred into 2 mL Eppendorf tubes and stored at −20 °C until analysis. Serum glucose, total protein, blood urea nitrogen (BUN), and beta-hydroxybutyrate (BHBA) analyses were performed at VETEST Veterinary Diagnostic and Analysis Laboratory (Bursa, Türkiye) using a Biosystems BA200 autoanalyzer (BioSystems S.A., Barcelona, Spain) and commercial BioSystems reagent kits (BioSystems S.A., Barcelona, Spain) according to the manufacturer’s instructions. Glucose was analyzed using the glucose oxidase/peroxidase method (COD 21503; endpoint monoreagent reaction; 505 nm with 670 nm reference). BUN was analyzed using the urease/glutamate dehydrogenase method (COD 21516; fixed-time bireagent reaction; 340 nm). Total protein was analyzed using the biuret method (COD 21513; differential bireagent reaction; 535 nm). BHBA was analyzed using the hydroxybutyrate dehydrogenase/diaphorase method (COD 21525; differential bireagent reaction; 560 nm).

### 2.5. Rumen Fluid Sampling and Analyses

Rumen fluid samples were collected on days 30 and 60, approximately 3–4 h after the morning feeding, using the same sampling procedure for all calves. Samples were obtained using a rubber rumen tube with an inner diameter of 2.5 cm and a length of 150 cm, and approximately 20–30 mL of rumen fluid was collected from each calf. To minimize saliva contamination, the first portion of rumen fluid was discarded and the subsequent sample was used for analysis. The pH of fresh rumen fluid was measured using a digital pH meter with 0.01 sensitivity (Knmaster, Istanbul, Turkey). The instrument was calibrated before measurements with standard buffer solutions (pH 4.00, 6.86, and 9.18). For volatile fatty acid analysis, rumen fluid was filtered through four layers of cheesecloth, and 1.5 mL of the filtrate was transferred into a tube containing a few drops of toluene to inhibit microbial activity [18]. The samples were stored in 2 mL Eppendorf tubes at −20 °C until analysis. Before analysis, the samples were thawed at room temperature and centrifuged at 5000 rpm for 10 min. Then, 120 µL of 25% metaphosphoric acid was added to 600 µL of the supernatant. From this mixture, 1 mL was transferred into vials, and volatile fatty acid analysis was performed by gas chromatography. The analyses were carried out using a Hewlett Packard Agilent Technologies 6890N system equipped with a 10% SP-1200/1% H_3_PO_4_ on 80/100 Chromosorb column. The detector temperature was set at 175 °C, the column temperature at 130 °C, and helium was used as the carrier gas.

### 2.6. Statistical Analysis

The data obtained in this study were analyzed using SPSS for Windows 25.0. Descriptive statistics are presented as mean ± standard deviation. Normality was assessed using Q–Q plots together with skewness and kurtosis values. Skewness and kurtosis values within the range of ±3 were considered acceptable for approximate normality. The choice of parametric or non-parametric tests was based on the distributional assessment of each variable. Variables that did not meet the normality assumption were analyzed using non-parametric tests. These included starter feed intake in weeks 1, 4, 5, and 6; BHBA on day 0; BUN on day 60; iso-butyric acid on days 30 and 60; butyric acid on days 30 and 60; iso-valeric acid on day 60; and n-valeric acid on day 60. For repeated measurements, including body weight and fecal score, repeated-measures ANOVA was used, with group, time, and group × time interaction included as fixed effects and calf treated as the repeated subject. When significant effects were detected, Tukey’s test was used for post hoc comparisons among independent treatment groups following one-way ANOVA when parametric assumptions were met. Bonferroni adjustment was used for multiple comparisons among repeated measurements and, where appropriate, for non-parametric multiple comparisons. For variables measured at two time points, paired-samples *t*-test or Wilcoxon signed-rank test was used for within-group comparisons, depending on data distribution. In addition, for variables measured at baseline and day 60, an additional covariate-adjusted analysis was performed using ANCOVA. For body weight on day 60, birth body weight was included as a covariate. For blood parameters on day 60, the corresponding day 0 value of each parameter was included as a covariate. This analysis was performed to evaluate whether group differences at day 60 remained significant after adjustment for baseline values. For comparisons among more than two independent groups, one-way ANOVA or Kruskal–Wallis test was used, depending on normality. For non-normally distributed repeated measurements, the Friedman test was used. In addition, selected outcomes were re-evaluated using two-way ANOVA within a 2 × 2 factorial structure. In this analysis, forage source (straw vs. alfalfa hay), method of offering (separate vs. mixed with starter feed), and forage source × method of offering interaction were included as fixed effects. The model used for this analysis was: Y = forage source + method of offering + forage source × method of offering + error. This factorial analysis was performed to distinguish the main effects of forage source and method of offering from their interaction. Statistical significance was accepted at *p* < 0.05.

## 3. Results

### 3.1. Body Weight and Average Daily Gain

Body weight increased significantly over time in all groups (*p* < 0.05). No significant differences were observed among groups on days 0, 15, 30, and 45 (*p* > 0.05), whereas a significant difference was detected on day 60 (*p* < 0.05). According to Tukey’s test, body weight in group A3 was higher than in group A1. When time, group, and group × time effects were evaluated together, the main effect of time and the group × time interaction were significant for body weight (*p* < 0.001), whereas the main effect of group was not significant (*p* = 0.454). These results indicate that body weight increased over time in all groups, but the pattern of increase differed among treatment groups. Body weight measurements by group are presented in Table 3, and the effects of time, group, and group × time on body weight are presented in Table 4.

No significant differences among groups were observed for ADG during days 0–15 (*p* > 0.05). During days 15–30, ADG was higher in group A3 than in group A1 (*p* = 0.048). During days 30–45, groups A3 and A4 had higher ADG than group A1 (*p* = 0.006). During days 45–60, groups A2 and A3 had higher ADG than group A1 (*p* = 0.002). Average daily gain values by group are presented in Table 5.

### 3.2. Starter Feed Intake

Weekly starter feed intake increased over time in all groups. No significant differences among groups were observed in weeks 1, 2, 3, 4, or 8 (*p* > 0.05). Significant group differences were detected in weeks 5, 6, and 7 (*p* < 0.001). In week 5, starter feed intake was higher in group A1 than in groups A3 and A4, and higher in group A2 than in group A4. In week 6, groups A1 and A2 had higher starter feed intake than group A4. In week 7, groups A1 and A2 had higher starter feed intake than groups A3 and A4. Weekly starter feed intake values are presented in Table 6.

### 3.3. Fecal Score

Fecal scores changed over time in all groups. No significant differences among groups were observed in weeks 1, 7, or 8 (*p* > 0.05), whereas significant group differences were detected in weeks 2, 3, 4, 5, and 6 (*p* < 0.05). In week 2, fecal score was higher in group A4 than in group A2. In weeks 3, 4, 5, and 6, group A4 had higher fecal scores than group A1. Weekly fecal score values by group are presented in Table 7.

### 3.4. Blood Parameters

Serum glucose concentrations decreased from day 0 to day 60 in groups A1, A2, and A4 (*p* < 0.05), whereas no significant change was observed in group A3 (*p* > 0.05). No significant difference among groups was observed on day 0 (*p* = 0.711); however, a significant group difference was detected on day 60 (*p* = 0.002), with group A3 having higher glucose concentration than group A1.

For total protein, no significant within-group changes were observed between day 0 and day 60 (*p* > 0.05). However, significant differences among groups were detected on both day 0 and day 60 (*p* < 0.05). On day 0, total protein concentration was higher in group A4 than in groups A1 and A2, and higher in group A3 than in group A2. On day 60, groups A2, A3, and A4 had higher total protein concentrations than group A1.

BHBA concentrations increased from day 0 to day 60 in groups A1, A2, and A4 (*p* < 0.05), whereas no significant change was observed in group A3 (*p* > 0.05). No significant differences among groups were observed for BHBA on either day 0 or day 60 (*p* > 0.05). For BUN, no significant within-group changes or between-group differences were detected (*p* > 0.05). Blood parameter values are presented in Table 8.

### 3.5. Rumen Parameters

Rumen pH increased from day 30 to day 60 in groups A2 and A4 (*p* < 0.05), whereas no significant change was observed in groups A1 and A3 (*p* > 0.05). No significant difference among groups was detected on day 30 (*p* = 0.369); however, a significant group difference was observed on day 60 (*p* = 0.003), with group A4 having higher rumen pH than groups A1 and A3.

No significant within-group changes or between-group differences were observed for acetic acid, propionic acid, iso-butyric acid, butyric acid, iso-valeric acid, n-valeric acid, or total VFA concentrations (*p* > 0.05). Rumen pH and volatile fatty acid values are presented in Table 9.

### 3.6. Correlation Analyses

A strong positive correlation was found between starter feed intake in the last week and average daily gain (r = 0.844; *p* < 0.01). This result indicates that calves with higher starter feed intake during the final week of the experimental period tended to have greater average daily gain. The results of this correlation analysis are presented in Table 10.

When the relationships between rumen and blood parameters on day 60 were examined, rumen pH was negatively correlated with acetic acid, propionic acid, butyric acid, n-valeric acid, and total volatile fatty acids (*p* < 0.01). These negative correlations indicate that lower rumen pH was associated with higher concentrations of several fermentation acids. In contrast, rumen pH was positively correlated with iso-valeric acid and total protein (*p* < 0.05). No significant correlations were found between rumen pH and glucose, BHBA, or BUN (*p* > 0.05). In general, strong positive correlations were observed among volatile fatty acids, suggesting that these fermentation end products varied in a coordinated manner. The results of the correlation analysis between rumen and blood parameters on day 60 are presented in Table 11.

### 3.7. Covariate-Adjusted Analysis

In the additional covariate-adjusted analysis, day 60 outcomes were evaluated after adjustment for the corresponding baseline values. The group effect remained significant for body weight on day 60 after adjustment for birth body weight (*p* = 0.003). For blood parameters, the group effect remained significant for serum glucose on day 60 after adjustment for day 0 glucose (*p* = 0.002) and for total protein on day 60 after adjustment for day 0 total protein (*p* = 0.005). In contrast, no significant group effect was detected for BHBA (*p* = 0.250) or BUN (*p* = 0.606) on day 60 after adjustment for their corresponding day 0 values.

### 3.8. Additional 2 × 2 Factorial Evaluation

To further distinguish the effects of forage source and method of offering, selected outcomes were re-evaluated using a two-way ANOVA within a 2 × 2 factorial structure. Forage source was compared as straw versus alfalfa hay, and method of offering was compared as separate offering versus mixing with starter feed. The forage source × method of offering interaction was also tested.

A significant forage source × method of offering interaction was detected for body weight on day 60 (*p* = 0.004). This interaction indicated that alfalfa hay was associated with higher final body weight when offered separately, whereas the same response was not observed when alfalfa hay was mixed with starter feed. Significant interactions were also detected for ADG during days 15–30 (*p* = 0.036) and days 45–60 (*p* = 0.002), showing that the effect of forage source on growth differed according to the method of offering.

Significant forage source effects were observed for ADG during days 30–45 (*p* = 0.001) and days 45–60 (*p* = 0.019), with higher values in calves receiving alfalfa hay than in those receiving straw. In contrast, starter feed intake during weeks 4–7 was affected by forage source (*p* ≤ 0.016), with higher values in the straw-fed groups than in the alfalfa-fed groups. Fecal score during weeks 2–7 was also affected by forage source (*p* ≤ 0.028), with higher scores in calves receiving alfalfa hay.

For blood parameters, serum glucose on day 60 showed both a forage source effect (*p* = 0.007) and a forage source × method of offering interaction (*p* = 0.004), indicating that the higher glucose concentration associated with alfalfa hay was mainly observed when alfalfa hay was offered separately. Total protein on day 60 was affected by both forage source (*p* < 0.001) and method of offering (*p* = 0.028), with higher values in calves receiving alfalfa hay and in calves receiving forage mixed with starter feed. BHBA on day 60 was affected by forage source (*p* = 0.027), with higher values in calves receiving straw than in those receiving alfalfa hay.

Rumen pH on day 60 was affected by method of offering (*p* = 0.001), with higher values in calves receiving forage mixed with starter feed than in those receiving forage separately. No significant forage source, method of offering, or interaction effects were observed for VFA concentrations on day 60 (*p* > 0.05). The results of the additional factorial analysis are presented in Supplementary Table S1.

## 4. Discussion

The findings of this study show that forage source and method of offering during the preweaning period may affect growth performance, rumen conditions, fecal consistency, and selected metabolic parameters in calves to different extents. The results also indicate that not all variables responded in the same direction. For this reason, forage use in early life should not be evaluated only as “forage included” or “forage not included”. Forage source, physical form, method of offering, starter feed characteristics, and calf age should be considered together. Previous studies have also emphasized that feeding strategies during the preweaning period should be evaluated by considering growth, digestive health, rumen development, and metabolic responses together, rather than by a single performance trait alone [19,20].

In terms of growth performance, the greater final body weight observed in group A3, despite the absence of the highest starter feed intake, indicates that growth performance was not driven by starter consumption alone. In this group, alfalfa hay was offered separately and had a higher crude protein content and better nutritive value than straw. This may have increased the overall quality of the solid feed consumed, even if measured starter intake was not the highest. A separate offering may also have allowed calves to consume forage according to individual preference, which could have improved diet selection and nutrient use. The better growth response in group A3 may therefore be related to forage quality and nutrient utilization rather than to starter intake alone. This interpretation is consistent with studies showing that alfalfa hay or higher-quality forage sources can influence growth performance, rumen fermentation, nutrient digestibility, and metabolic responses in Holstein calves [13,21,22]. Because individual forage intake was not recorded in the separately offered forage groups, this explanation should be interpreted cautiously. The additional 2 × 2 factorial analysis supported this interpretation by showing a significant forage source × method of offering interaction for final body weight. This indicates that the effect of alfalfa hay on growth depended on how it was offered, with the most favorable response observed when alfalfa hay was provided separately.

The higher body weight observed in group A3 should not be interpreted only as a short-term performance outcome. Greater average daily gain during the preweaning period has been associated with later productive performance, suggesting that early growth may have longer-term biological relevance [19]. The present study was limited to the preweaning period, and no postweaning or lactation performance data were collected. The long-term implications of the observed growth differences remain to be confirmed in future studies.

The effect of the forage-offering method has not been consistent across studies. Engelking et al. [9] reported that offering forage mixed with starter feed or separately did not create marked differences in some performance traits during the preweaning and weaning periods, although differences became more apparent after weaning. Antúnez-Tort et al. [14] who compared barley straw and alfalfa hay, found no clear difference in total dry matter intake during the preweaning period. These findings indicate that the response to forage provision depends not only on forage source, but also on physical form, method of offering, starter feed characteristics, and timing of application.

One notable finding of the present study is that starter feed intake and growth performance did not always follow the same pattern. Although higher starter intake was observed in the straw-fed groups during some weeks, the greatest final body weight was recorded in group A3. The mismatch between starter feed intake and final body weight agrees with meta-analytical findings showing that the response to forage feeding in preweaned calves depends on forage source, physical form, offering method, and starter feed characteristics, not only on the amount of solid feed [19,23]. Xiao et al. [23] reported that forage feeding can affect growth performance, rumen fermentation, and nutrient digestibility, but that the direction and magnitude of these responses vary according to how forage is supplied. In the present study, the straw-fed groups had higher starter intake during some weeks, whereas group A3 had the highest final body weight. This pattern suggests that feed quality, rumen conditions, and nutrient utilization may have contributed more to growth than starter intake alone. The present study contributes to the existing literature by evaluating forage source and method of offering within the same experimental framework and by interpreting their distinct associations with growth performance and rumen conditions. In this study, separately offered alfalfa hay was more closely associated with improved growth performance, whereas chopped alfalfa hay mixed with starter feed was more closely associated with higher rumen pH. This distinction may help explain why previous studies have reported variable responses to forage provision in preweaned calves.

For rumen parameters, the highest pH value on day 60 was observed in group A4, in which chopped alfalfa hay was mixed with starter feed. The higher rumen pH observed in this group, despite the absence of significant differences in VFA concentrations, suggests that the pH response was more likely related to buffering conditions than to reduced fermentation. Chopped alfalfa hay mixed with starter feed may have increased the physical effectiveness of the diet by providing structural fiber together with concentrate intake. This could have stimulated chewing activity and salivary secretion, increasing the supply of bicarbonate to the rumen. Such buffering can increase rumen pH without necessarily reducing VFA production or changing the measured VFA profile. Similar responses have been reported in studies showing that forage provision can support rumen pH through physical fiber effects and salivary buffering, while VFA concentrations do not always change in parallel [3,12,24]. The additional 2 × 2 factorial analysis also showed a significant method of offering effect for rumen pH on day 60, with higher values in calves receiving forage mixed with starter feed. This supports the interpretation that mixing chopped forage with starter feed may have improved ruminal buffering conditions. Since chewing behavior and diurnal rumen pH were not measured, this mechanism should be considered a plausible explanation rather than direct evidence.

The correlation findings also support the relationship between solid feed intake, fermentation activity, and rumen conditions. The strong positive correlation between last-week starter feed intake and average daily gain indicates that calves with greater starter intake during the final week of the experimental period tended to have higher growth performance. This finding supports the importance of solid feed consumption for growth during the late preweaning period. Starter feed intake is a major driver of ruminal fermentation and volatile fatty acid production, which provide important energy substrates and contribute to ruminal epithelial development and body weight gain [7,23]. Because individual forage intake was not recorded in the separately offered forage groups, this relationship should be interpreted as an association between starter intake and growth rather than total dry matter intake and growth.

The negative correlations observed between rumen pH and acetic acid, propionic acid, butyric acid, n-valeric acid, and total VFA on day 60 reflect the expected inverse relationship between fermentation acid accumulation and ruminal pH. As ruminal fermentation activity increases, VFA accumulation can contribute to a decline in rumen pH unless sufficiently buffered by saliva secretion and ruminal absorption [3,12]. The positive correlation between rumen pH and total protein, together with the negative correlation between total protein and BUN, may indicate an association between rumen conditions and systemic protein- or nitrogen-related metabolites. These relationships should not be interpreted as direct evidence of improved microbial protein synthesis, because microbial protein flow, nitrogen balance, and urinary nitrogen excretion were not measured in the present study. These correlations are presented as exploratory findings that may help guide future studies on the relationship between rumen environment and blood metabolite profiles in preweaned calves.

Among the blood parameters, the glucose and total protein findings deserve particular attention. The higher glucose concentration observed in group A3 on day 60 suggests that some differences in energy metabolism may have existed among groups. The additional covariate-adjusted analysis showed that the day 60 difference in glucose remained significant after adjustment for day 0 glucose, supporting the relevance of this finding. Nevertheless, glucose concentration should be interpreted as a general metabolic indicator rather than as direct evidence of rumen development or improved energy utilization. Jafari et al. [25] reported that, as solid feed intake increases with age, the relative importance of glucose decreases and volatile fatty acids produced in the rumen become more important. The increase in BHBA observed in some groups may also be considered a physiological reflection of the transition toward greater solid feed utilization and ruminal epithelial development. Kazemi-Bonchenari et al. [26] reported that BHBA may vary in relation to starter intake and ruminal fermentative activity, whereas glucose and urea-related metabolites do not always respond in the same direction.

The lack of significant differences in BUN suggests that forage source and method of offering had a limited effect on nitrogen-related blood metabolites under the conditions of this study. Total protein results should also be interpreted carefully because of sampling time and baseline variation. Total protein measured during the first 24–36 h after birth is related more closely to colostrum intake and passive transfer than to the experimental diet. Lombard et al. [27] reported that serum total protein can be used to evaluate passive transfer in the first days of life, and Godden et al. [28] emphasized the importance of colostrum management for calf health and vitality. In the present study, the additional covariate-adjusted analysis showed that the day 60 group difference in total protein remained significant after adjustment for day 0 total protein. This supports the robustness of the day 60 difference, but it does not identify the underlying mechanism. Because microbial protein synthesis, nitrogen balance, immunoglobulin concentrations, and urinary nitrogen excretion were not measured, the total protein findings should be interpreted as an indicator of systemic protein status rather than direct evidence of improved nitrogen utilization. Differences in total protein observed at the beginning of the study should not be attributed directly to the experimental treatments. Differences observed on day 60 may be more closely related to nutritional status and metabolic adaptation.

The fecal score results may also have practical relevance for digestive stability and calf welfare. Higher fecal scores in group A4 during weeks 2 to 6 indicate that calves receiving chopped alfalfa hay mixed with starter feed had looser feces during part of the early preweaning period. This may reflect a more variable gastrointestinal adaptation to the simultaneous intake of starter feed and chopped forage. Changes in digesta consistency, passage dynamics, and nutrient digestion may contribute to such temporary fecal changes. The absence of significant differences in weeks 7 and 8 suggests that this response was transient rather than persistent. Fecal score should therefore be interpreted together with growth performance, rumen fermentation, and blood metabolites, rather than as a standalone indicator of gut health. From a welfare perspective, monitoring fecal consistency remains important because persistent loose feces may impair hydration, comfort, and nutrient utilization in young calves. Fecal and health responses in preweaned calves are also influenced by management, colostrum status, environment, and diet composition [19,26,28].

Overall, the present findings indicate that growth performance, starter feed intake, rumen pH, VFA concentrations, fecal score, and blood metabolites should be interpreted together rather than as independent outcomes. The higher final body weight observed in calves receiving alfalfa hay separately occurred despite lower starter intake during some weeks, suggesting that forage quality and nutrient use may have contributed to growth beyond starter consumption alone. In contrast, the higher rumen pH observed when chopped alfalfa hay was mixed with starter feed was not accompanied by significant differences in VFA concentrations, suggesting that the pH response may have been related more to buffering conditions than to reduced fermentation. These divergent responses help explain why previous studies have reported variable effects of forage provision in preweaned calves. Differences in forage source, physical form, method of offering, starter feed characteristics, calf age, and sampling time may all contribute to differences among studies.

When interpreting these findings, several methodological considerations should be taken into account. Although calves were allocated in a sex-balanced manner, sex was not included as a fixed factor in the primary statistical model; therefore, possible sex-related variation should be considered when interpreting the results. Rumen fluid samples were collected only on days 30 and 60 and at a single post-feeding interval after the morning feeding; therefore, the results may not fully represent diurnal variation in rumen pH and VFA concentrations. Individual forage intake was not recorded in the separately offered forage groups because reliable measurement of forage refusals was not possible under outdoor hutch conditions. Total dry matter intake and individual forage consumption patterns could not be determined. Finally, the geometric mean particle size of the chopped straw and alfalfa hay was not determined using a standardized particle-size method; thus, the reported chop length should be interpreted as an approximate physical description rather than a measured particle-size distribution. Despite these limitations, the study provides useful comparative information on the effects of forage source and offering method on growth performance, rumen conditions, fecal consistency, and selected blood parameters in preweaned calves.

## 5. Conclusions

This study showed that forage source and method of offering during the preweaning period produced different responses in selected performance, rumen, fecal score, and blood parameters. Under the conditions of the present study, offering alfalfa hay separately from starter feed was associated with higher final body weight and higher average daily gain during selected periods. In contrast, mixing chopped alfalfa hay with starter feed was associated with higher rumen pH on day 60, whereas VFA concentrations were not significantly altered among groups. Starter feed intake was higher mainly in the straw-fed groups during weeks 5–7, indicating that higher starter intake did not necessarily correspond to higher final body weight.

From a practical feeding perspective, separately offered alfalfa hay may be considered a useful option when the objective is to support final body weight gain in preweaned calves, whereas chopped alfalfa hay mixed with starter feed may be useful when the objective is to support rumen pH. These implications should be applied cautiously under farm conditions, because individual forage intake was not recorded, total dry matter intake could not be determined, sex was not included as a fixed factor in the primary statistical model, and calves were monitored only until day 60. Post-weaning growth and long-term productive responses were not evaluated. Future studies should include individual forage intake, post-weaning performance, behavioral measurements, sex as a model factor where possible, and longer follow-up periods to better define practical feeding recommendations for dairy calf rearing.

## Figures and Tables

**Table 1:** Nutrient composition of the milk replacer and starter feed used in the experiment, as-fed basis.

Parameter (%)	Milk Replacer	Starter Feed
Crude protein	23.00	18.00
Crude fat	18.00	3.60
Crude ash	8.00	7.60
Crude fiber	0.00	7.40
Calcium	0.80	1.00
Sodium	0.60	0.30
Phosphorus	0.80	0.80

Values are expressed on an as-fed basis according to product label or ration formulation information.

**Table 2:** Nutrient composition of the forages used in the experiment, dry matter basis.

Forage	Dry Matter	Crude Protein	Crude Fiber	Crude Ash	Crude Fat	Starch	NEL (kcal/kg)	NDF	ADF	ADL
Alfalfa hay	100.00	17.45	23.44	9.66	1.85	0.00	1290.61	46.76	35.43	9.25
Straw	100.00	2.56	46.31	8.18	0.54	0.00	774.48	64.63	54.92	10.77

Values are expressed on a dry matter basis. NEL, net energy for lactation; NDF, neutral detergent fiber; ADF, acid detergent fiber; ADL, acid detergent lignin.

**Table 3:** Body weight measurements by group, kg.

Group	Day 0 (1)	Day 15 (2)	Day 30 (3)	Day 45 (4)	Day 60 (5)
A1	39.89 ± 4.20	41.08 ± 4.35	45.37 ± 6.04	54.52 ± 5.03	65.82 ± 6.41 ^b^
A2	39.77 ± 3.82	40.39 ± 5.01	46.39 ± 5.99	57.62 ± 7.49	72.18 ± 5.99 ^ab^
A3	38.20 ± 3.71	39.25 ± 4.72	48.37 ± 7.13	59.78 ± 8.04	73.37 ± 7.87 ^a^
A4	36.90 ± 1.79	38.11 ± 2.23	42.71 ± 3.33	59.14 ± 3.66	67.93 ± 3.40 ^ab^
*p*-value	0.197	0.424	0.194	0.265	0.030

Values are presented as mean ± SD. Different superscript letters within the same column indicate significant differences among groups (*p* < 0.05). Groups sharing at least one common letter are not significantly different. The *p*-value row indicates the significance of between-group comparisons at each time point.

**Table 4:** Effects of time, group, and group × time on body weight.

Effect	F-Value	*p*-Value	η^2^
Time	901.465	<0.001	0.962
Group × time	5.602	<0.001	0.318
Group	0.894	0.454	0.069

Repeated measures ANOVA was used to evaluate the effects of time, group, and group × time interaction on body weight. Calf was treated as the repeated subject. η^2^: partial eta squared.

**Table 5:** Average daily gain values by group, kg/day.

Group	ADG1	ADG2	ADG3	ADG4
A1	0.08 ± 0.10	0.18 ± 0.13 ^b^	0.33 ± 0.10 ^b^	0.43 ± 0.09 ^b^
A2	0.04 ± 0.14	0.22 ± 0.14 ^ab^	0.40 ± 0.14 ^ab^	0.54 ± 0.09 ^a^
A3	0.07 ± 0.18	0.34 ± 0.16 ^a^	0.48 ± 0.13 ^a^	0.59 ± 0.10 ^a^
A4	0.08 ± 0.10	0.19 ± 0.10 ^ab^	0.49 ± 0.06 ^a^	0.52 ± 0.04 ^ab^
*p*-value	0.907	0.048	0.006	0.002

Values are presented as mean ± SD. ADG1–ADG4 represent days 0–15, 15–30, 30–45, and 45–60, respectively. Different superscript letters within the same column indicate significant differences according to Tukey’s post hoc test (*p* < 0.05). Groups sharing a common letter are not significantly different.

**Table 6:** Weekly starter feed intake values by group.

Group	Week 1	Week 2	Week 3	Week 4	Week 5	Week 6	Week 7	Week 8
A1	10.8 ± 6.36	28.5 ± 22.24	157.8 ± 149.91	437.5 ± 239.23	683.5 ± 212.30 ^a^	836.0 ± 248.62 ^a^	1052.7 ± 259.62 ^a^	1263.5 ± 308.15
A2	8.5 ± 7.31	44.2 ± 59.01	241.0 ± 159.35	413.0 ± 186.13	602.0 ± 149.28 ^ab^	767.0 ± 145.99 ^a^	984.0 ± 175.76 ^a^	1349.0 ± 273.19
A3	11.0 ± 14.87	81.0 ± 76.77	176.0 ± 123.31	303.0 ± 81.52	459.5 ± 66.69 ^bc^	610.5 ± 121.35 ^ab^	759.5 ± 144.35 ^b^	1220.0 ± 226.32
A4	2.9 ± 3.18	26.0 ± 56.80	151.0 ± 89.99	276.0 ± 130.66	391.0 ± 98.48 ^c^	546.5 ± 62.36 ^b^	746.0 ± 83.29 ^b^	1151.0 ± 118.27
*p*-value	0.054	0.137	0.428	0.073	<0.001	<0.001	<0.001	0.334

Values are presented as mean ± SD. Between-group comparisons were performed using one-way ANOVA or Kruskal–Wallis test depending on data distribution. Different superscript letters within the same column indicate significant differences among groups after multiple comparisons (*p* < 0.05). Groups sharing a common letter are not significantly different.

**Table 7:** Weekly fecal score values by group.

Group	FS1	FS2	FS3	FS4	FS5	FS6	FS7	FS8
A1	2.8 ± 0.63	2.4 ± 0.52 ^ab^	1.8 ± 0.42 ^b^	1.6 ± 0.52 ^b^	1.5 ± 0.53 ^b^	1.3 ± 0.48 ^b^	1.3 ± 0.48	1.4 ± 0.52
A2	2.1 ± 0.99	2.1 ± 0.88 ^b^	2.3 ± 0.67 ^ab^	1.9 ± 0.74 ^ab^	1.9 ± 0.88 ^ab^	1.5 ± 0.53 ^ab^	1.3 ± 0.48	1.4 ± 0.70
A3	2.9 ± 0.99	3.2 ± 1.55 ^ab^	2.9 ± 1.52 ^ab^	2.4 ± 1.07 ^ab^	2.1 ± 0.74 ^ab^	2.1 ± 0.99 ^ab^	1.7 ± 0.67	1.6 ± 0.70
A4	2.8 ± 0.92	3.25 ± 0.92 ^a^	3.2 ± 1.32 ^a^	2.7 ± 1.06 ^a^	2.5 ± 0.85 ^a^	2.2 ± 0.63 ^a^	1.8 ± 0.79	1.7 ± 0.67
*p*-value	0.185	0.038	0.030	0.037	0.043	0.013	0.166	0.665

Values are presented as mean ± SD. FS1–FS8 represent weekly fecal score measurements from week 1 to week 8, respectively. Fecal scores were defined as follows: 1 = firm/normal feces, 2 = soft feces with a distinct form, 3 = soft feces with no clear form, 4 = watery feces, and 5 = watery feces containing blood and/or mucus. Between-group comparisons were performed using one-way ANOVA. Different superscript letters within the same column indicate significant differences among groups according to Tukey’s post hoc test (*p* < 0.05). Groups sharing a common letter are not significantly different.

**Table 8:** Serum glucose, total protein, BHBA, and BUN values by group.

Parameter	Group	Day 0	Day 60	Within-Group *p*-Value
Glucose, mg/dL	A1	123.8 ± 45.57	83.6 ± 10.01 ^b^	0.012
	A2	140.4 ± 19.83	92.7 ± 9.31 ^ab^	<0.001
	A3	127.9 ± 24.90	104.3 ± 15.51 ^a^	0.069
	A4	134.0 ± 38.39	91.9 ± 7.59 ^ab^	0.004
Between-group *p*-value		0.711	0.002	
Total protein, g/dL	A1	6.52 ± 0.93 ^bc^	6.06 ± 0.54 ^b^	0.103
	A2	6.27 ± 1.26 ^c^	6.97 ± 0.35 ^a^	0.077
	A3	7.83 ± 0.82 ^ab^	7.46 ± 1.25 ^a^	0.395
	A4	8.26 ± 1.54 ^a^	7.63 ± 0.51 ^a^	0.141
Between-group *p*-value		0.001	<0.001	
BHBA, mg/dL	A1	0.67 ± 0.22	2.07 ± 0.81	0.005
	A2	0.65 ± 0.13	2.47 ± 0.84	0.005
	A3	1.02 ± 0.68	1.60 ± 0.74	0.203
	A4	0.87 ± 0.37	1.79 ± 0.73	0.014
Between-group *p*-value		0.338	0.091	
BUN, mg/dL	A1	7.18 ± 2.91	7.16 ± 1.47	0.646
	A2	7.53 ± 2.49	6.43 ± 2.11	0.285
	A3	6.80 ± 1.82	6.77 ± 5.33	0.241
	A4	4.90 ± 2.17	5.48 ± 1.74	0.508
Between-group *p*-value		0.082	0.098	

Values are presented as mean ± SD. Different superscript letters within the same column and parameter indicate significant differences among groups after multiple comparisons (*p* < 0.05). Groups sharing a common letter are not significantly different. Within-group *p*-values indicate comparisons between day 0 and day 60. Glucose, BHBA, and BUN are expressed as mg/dL, and total protein is expressed as g/dL. BHBA: beta-hydroxybutyrate; BUN: blood urea nitrogen.

**Table 9:** Rumen pH and volatile fatty acid values by group.

Parameter	Group	Day 30	Day 60	Within-Group *p*-Value
pH	A1	5.85 ± 0.45	5.87 ± 0.43 ^b^	0.915
	A2	5.78 ± 0.35	6.20 ± 0.32 ^ab^	0.014
	A3	5.88 ± 0.48	6.02 ± 0.32 ^b^	0.399
	A4	5.59 ± 0.25	6.49 ± 0.35 ^a^	<0.001
Between-group *p*-value		0.369	0.003	
Acetic acid, mmol/L	A1	10.80 ± 4.21	12.78 ± 3.00	0.290
	A2	12.11 ± 3.50	12.61 ± 4.88	0.810
	A3	14.27 ± 6.05	13.06 ± 5.76	0.703
	A4	13.37 ± 4.91	10.47 ± 7.34	0.308
Between-group *p*-value		0.399	0.702	
Propionic acid, mmol/L	A1	9.19 ± 5.58	11.86 ± 3.16	0.277
	A2	11.78 ± 5.80	12.28 ± 7.36	0.869
	A3	14.62 ± 7.61	12.64 ± 9.07	0.631
	A4	13.64 ± 7.37	10.16 ± 10.64	0.441
Between-group *p*-value		0.294	0.906	
Iso-butyric acid, mmol/L	A1	0.13 ± 0.06	0.15 ± 0.04	0.333
	A2	0.15 ± 0.07	0.17 ± 0.07	0.333
	A3	0.22 ± 0.17	0.23 ± 0.26	0.721
	A4	0.15 ± 0.09	0.24 ± 0.20	0.114
Between-group *p*-value		0.445	0.574	
Butyric acid, mmol/L	A1	5.02 ± 3.31	6.58 ± 1.90	0.203
	A2	7.06 ± 3.40	7.04 ± 4.18	0.959
	A3	9.08 ± 5.99	8.80 ± 11.25	0.575
	A4	8.85 ± 5.35	4.83 ± 3.70	0.093
Between-group *p*-value		0.124	0.337	
Iso-valeric acid, mmol/L	A1	0.23 ± 0.11	0.24 ± 0.09	0.878
	A2	0.27 ± 0.12	0.27 ± 0.10	0.878
	A3	0.29 ± 0.17	0.38 ± 0.39	0.959
	A4	0.26 ± 0.16	0.40 ± 0.20	0.059
Between-group *p*-value		0.806	0.184	
n-Valeric acid, mmol/L	A1	0.90 ± 0.93	1.37 ± 0.52	0.114
	A2	1.06 ± 0.59	1.29 ± 0.85	0.575
	A3	2.01 ± 1.43	1.82 ± 2.68	0.508
	A4	1.13 ± 0.58	0.74 ± 0.72	0.169
Between-group *p*-value		0.055	0.273	
Total VFA, mmol/L	A1	26.28 ± 13.55	32.98 ± 6.62	0.233
	A2	32.42 ± 12.32	33.66 ± 16.55	0.856
	A3	40.49 ± 19.00	36.93 ± 25.38	0.775
	A4	37.40 ± 15.39	26.84 ± 22.11	0.260
Between-group *p*-value		0.195	0.693	

Different superscript letters within the same column and parameter indicate significant differences among groups after multiple comparisons (*p* < 0.05). Groups sharing a common letter are not significantly different. Within-group *p*-values indicate comparisons between day 30 and day 60. Volatile fatty acid concentrations are expressed as mmol/L; pH is unitless. VFA: volatile fatty acids.

**Table 10:** Correlation analysis between starter feed intake in the last week and average daily gain.

Variables	1	2
1. Last week’s starter feed intake	1	
2. Average daily gain	0.844 **	1

** *p* < 0.01. Pearson correlation was used.

**Table 11:** Correlation analysis between rumen and blood parameters on day 60.

Variables	1	2	3	4 ^s^	5 ^s^	6 ^s^	7 ^s^	8	9	10	11	12
1. pH	1											
2. Acetic acid	−0.510 **	1										
3. Propionic acid	−0.557 **	0.932 **	1									
4. Iso-butyric acid ^s^	0.094	0.089	0.123	1								
5. Butyric acid ^s^	−0.534 **	0.830 **	0.850 **	0.252	1							
6. Iso-valeric acid ^s^	0.326 *	0.025	0.080	0.586 **	0.129	1						
7. n-Valeric acid ^s^	−0.565 **	0.795 **	0.849 **	0.105	0.908 **	0.014	1					
8. Total VFA	−0.516 **	0.934 **	0.913 **	0.171	0.910 **	0.084	0.890 **	1				
9. Glucose	0.044	0.055	0.114	0.071	−0.060	0.185	0.044	0.174	1			
10. Total protein	0.360 *	−0.064	−0.072	−0.033	−0.263	0.178	−0.308	−0.104	−0.004	1		
11. BHBA	0.020	0.059	−0.037	0.099	0.285	−0.160	0.161	0.083	−0.180	−0.077	1	
12. BUN ^s^	0.013	−0.167	−0.153	0.007	−0.115	0.093	−0.006	−0.127	0.011	−0.351 *	−0.037	1

* *p* < 0.05; ** *p* < 0.01. Variables marked with superscript “s” were analyzed using Spearman correlation; all other variables were analyzed using Pearson correlation. VFA: volatile fatty acids; BHBA: beta-hydroxybutyrate; BUN: blood urea nitrogen.

## Data Availability

The raw data supporting the findings of this study are provided in the Supplementary Materials.

## Supplementary Materials

The following supporting information can be downloaded at: https://www.mdpi.com/article/10.3390/ani16101462/s1. Table S1: Results of the additional 2 × 2 factorial analysis evaluating forage source, method of offering, and their interaction; Supplementary Dataset S1: Anonymized raw data used in the study.
