# Supplementary material for: Effects of Forage Source and Method of Offering on Growth Performance, Starter Feed Intake, Rumen Fermentation, and Selected Blood Parameters in Preweaned Holstein Calves
Source: Animals (Basel). 2026 May 10;16(10):1462. doi: 10.3390/ani16101462 (PMC13203234; doi:10.3390/ani16101462)
Supplement: Supplementary file 1 [file animals-16-01462-s001.zip › Supplementary_Material Table S1.pdf]

Supplementary Table S1. Results of the additional 2 × 2 factorial analysis evaluating forage source, method of offering, and their interaction.

Variable	Forage source effect	Direction	Method effect	Direction	Interaction	Direction / interpretation
Body weight, day 60	0.401	—	0.814	—	**0.004**	Alfalfa hay increased final BW when offered separately, but not when mixed
ADG, days 15–30	0.134	—	0.211	—	**0.036**	Alfalfa-separate had the highest ADG
ADG, days 30–45	**0.001**	Alfalfa > straw	0.230	—	0.426	Main forage source effect
ADG, days 45–60	**0.019**	Alfalfa > straw	0.470	—	**0.002**	Response depended on offering method
Starter intake, week 4	**0.016**	Straw > alfalfa	0.635	—	0.982	Main forage source effect
Starter intake, week 5	**<0.001**	Straw > alfalfa	0.105	—	0.886	Main forage source effect
Starter intake, week 6	**<0.001**	Straw > alfalfa	0.196	—	0.961	Main forage source effect
Starter intake, week 7	**<0.001**	Straw > alfalfa	0.469	—	0.626	Main forage source effect

Variable	Forage source effect	Direction	Method effect	Direction	Interaction	Direction / interpretation
Fecal score, week 2	**0.005**	Alfalfa > straw	0.705	—	0.596	Main forage source effect
Fecal score, week 3	**0.006**	Alfalfa > straw	0.250	—	0.772	Main forage source effect
Fecal score, week 4	**0.007**	Alfalfa > straw	0.288	—	1.000	Main forage source effect
Fecal score, week 5	**<0.001**	Alfalfa > straw	**0.003**	Mixed > separate	0.118	Main forage and method effects
Fecal score, week 6	**0.001**	Alfalfa > straw	0.496	—	0.820	Main forage source effect
Fecal score, week 7	**0.028**	Alfalfa > straw	0.801	—	0.801	Main forage source effect
Glucose, day 60	**0.007**	Alfalfa > straw	0.638	—	**0.004**	Higher glucose with alfalfa was mainly observed when offered separately
Total protein, day 60	**<0.001**	Alfalfa > straw	**0.028**	Mixed > separate	0.132	Main forage and method effects
BHBA, day 60	**0.027**	Straw > alfalfa	0.233	—	0.682	Main forage source effect

Variable	Forage source effect	Direction	Method effect	Direction	Interaction	Direction / interpretation
Rumen pH, day 60	0.057	—	**0.001**	Mixed > separate	0.527	Main method effect
VFA variables, day 60	>0.05	—	>0.05	—	>0.05	No factorial effect

Values represent p-values from two-way ANOVA. Forage source was compared as straw versus alfalfa hay, and method of offering was compared as separate offering versus mixing with starter feed. The interaction term represents forage source × method of offering. Direction indicates the factor level with the higher mean when the corresponding main effect was significant. For variables with significant interaction effects, interpretation was based on treatment-group means. ADG: average daily gain; BHBA: beta-hydroxybutyrate; VFA: volatile fatty acids. Significant effects are shown in bold.
